# Circular Dichroism Microscopy Free from Commingling Linear Dichroism via Discretely Modulated Circular Polarization

**DOI:** 10.1038/srep35731

**Published:** 2016-10-20

**Authors:** Tetsuya Narushima, Hiromi Okamoto

**Affiliations:** 1 Institute for Molecular Science and The Graduate University for Advanced Studies (Sokendai), 38 Nishigonaka, Myodaiji, Okazaki, Aichi 444-8585, JAPAN; 2 PRESTO, Japan Science and Technology Agency, 4-1-8 Honcho, Kawaguchi, Saitama, 332-0012, JAPAN

## Abstract

In this work, we developed a circular dichroism (CD) imaging microscope with a device to suppress the commingling of linear birefringence (LB) and linear dichroism (LD) signals. CD signals are, in principle, free from the commingling influence of LD and LB if the sample is illuminated with pure circularly polarized light, with no linear polarization contribution. Based on this idea, we here propose a novel circular polarization modulation method to suppress the contribution of linear polarization, which enables high-sensitivity CD detection (10^−4^ level in optical density unit or mdeg level in ellipticity) for microscopic imaging at a nearly diffraction limited spatial resolution (sub-μm level). The highly sensitive, diffraction-limited local CD detection will make direct analyses of chiral structures and spatial mappings of optical activity feasible for μm- to sub-μm-sized materials and may yield a number of applications as a unique optical imaging method.

Molecules with no improper rotation axis (chiral molecules) exhibit optical activities, which are typified by optical rotation or circular dichroism (CD) and play a crucial role in materials and biological sciences. Whereas exact molecular structures can be determined by X-ray crystallography, if crystalline samples are available, spectroscopic techniques based on optical activity are widely used to analyse the structural asymmetry of molecules or the secondary structures of protein molecules dispersed in water solution. The optical activities associated with three-dimensional molecules, including large molecules and proteins, originate from the rotational strength, which is defined as an inner product of electric and magnetic transition dipole moments. Although this mechanism of optical activity is attributable to point transition dipoles, the optical activity of spatially extended (nanostructured) chiral systems, in which other mechanisms may contribute, is also possible, and optical activity of this type has been observed in some systems^1^,^2^,^3^. Some types of interaction between light and matter become efficient as the size of the material approaches the wavelength of light; optical activity typifies such an interaction. Optical activity becomes prominent when the spatial scale of chirality of the material is close to the wavelength of light. Although achiral molecules do not exhibit any optical activity when they are isolated from each other, some aggregates of achiral molecules show optical activity because of the formation of mesoscopically chiral structures^2^.

In a macroscopic (or classical electromagnetic) picture, optical activity arises from the difference between the complex refractive index for left circularly polarized (LCP) light and that for right circularly polarized (RCP) light. Optical rotation (OR), also referred to as circular birefringence (CB), is attributed to the difference in the real part of the complex refractive index and involves the rotation of the polarization plane of incident linearly polarized (LP) light. In contrast, CD stems from the difference in the imaginary part of the complex refractive index and converts the incident linear polarization into elliptical polarization. Compared with linear birefringence (LB) and linear dichroism (LD), which originate in the anisotropy of materials against linear polarization, the signal intensities of CB and CD, which involve an optical response to circular polarization, are usually two or three orders of magnitude smaller^4^,^5^. In commercial CD spectrometers (Fig. 1a)^6^, polarization modulation method with a photoelastic modulator (PEM) is commonly adopted to enhance sensitivity. During the modulation, the polarization continuously changes from left circular polarization (LCPn) to right circular polarization (RCPn) through linear polarization (LPn). Therefore, the LP component in a particular direction is involved in the polarization modulation at any time. Various non-ideal properties of the apparatus, such as the non-linear response of optical elements, residual strain in the light modulation device, and higher harmonic responsiveness of the lock-in amplifier, complicate the exact synchronization of modulation between RCPn and LCPn to lock-in detection. As a result, an artefact signal resulting from the LP components is commingled in the signal^7^,^8^. In CD measurements, one must pay careful attention to commingling of the false signals arising from LB and LD, unless the sample is isotropic.

In CD measurements for anisotropic samples, e.g., crystalline materials, the samples are occasionally rotated in the observing plane (normal to the direction of light incidence) to suppress the influence of the LP component. This sample rotation method is effective only if the sample is uniform over the area of observation. Recently, alternative methods to suppress the LPn influence were examined. Using a spinning half waveplate, the LP components were rotated with time, which allows the LD and LB contribution to be cancelled out if the data acquisition time is sufficiently long^9^. A full analysis of all of the polarization states macroscopically caused by the anisotropy and chirality of a sample was demonstrated^10^. Similarly, a microscopic approach to analyze all Müller matrix elements, including CD components, has been proposed^11^. Another approach was also proposed for the complete elimination of the influence of LP component by obtaining the difference between the transmitted light intensities measured separately for LCP and RCP incidence (we call this method the discrete illumination of circularly polarized light (CPL) in this paper), which was applied for a microscopic CD measurement^12^. In those microscopic cases, the advantage of polarization modulation with lock-in detection, which potentially improve the detection sensitivity, was not exploited.

In this work, we constructed a microscope specialised for imaging with CD that is equipped with a device used to avoid the commingling of the LD and LB signals. The CD signal is, in principle, free from the influence of the commingling of the LD and LB signals if the sample is illuminated only with highly pure CPL. We developed a high-sensitivity detection method based on this approach. In addition to this device, the introduction of a laser as a light source with high brightness and small beam divergence and the detailed tuning of the surrounding environment enabled us to achieve high CD sensitivities of 10^−4^ level in the optical density unit (mdeg level in ellipticity) at a nearly diffraction limited spatial resolution (sub-μm level). Because the local CD distribution is sensitively observed with high spatial resolution, we can conduct, for example, direct analyses of the chiral structures in materials at the spatial scale of μm to sub-μm as well as spatial mappings of optical activity in polymer thin films and liquid crystals. The CD microscope proposed here may yield many potential applications based on the unique optical imaging method.

## Experimental Arrangement

### Optical system for discretely modulated circular polarization

**Figure 1:**
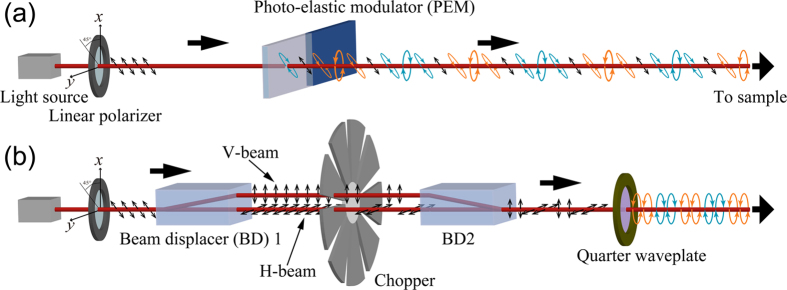
Preparation of modulated CPL beams that periodically alternate between LCPn and RCPn. (**a**) CPL modulation with a PEM adopted in commercial CD spectrometers. (**b**) Discrete CPL modulation with a pair of polarization BDs proposed in this study. The modulated light with the PEM in (**a**) unavoidably involves LP components when the polarization transits between LCPn and RCPn. In the discrete modulation in (**b**), the BD1 divides an incident LP beam, with a polarization angle of 45 degrees from the *y*-axis, into horizontally and vertically polarized beams. A rotatory chopper blade with a 25% duty cycle always blocks one of the split two beams, and horizontally and vertically polarized beams are alternately delivered to the subsequent optical system. The split beams are recombined with another BD into one beam. By converting the modulated light beam into CPL through a quarter waveplate, the discrete CPL modulation is obtained, for which the LP components are not involved.

**Figure 2:**
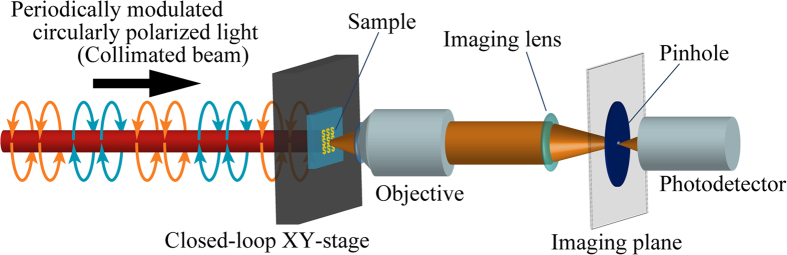
Transmission-mode CD microscope with sample scanning. To maintain the high purity of the incident polarization, a collimated beam of periodically modulated light between LCPn and RCPn was used for illumination of the sample. The optical responses (CD signals) on the sample surface form an image on the image plane through an objective lens (NA 0.75) and an imaging lens (#58-520, Edmund Optics Inc.). If a pinhole is placed on the image plane, a local optical response at a specific small area of the sample can be selectively detected. By scanning the sample, the spatial distribution of the local optical response is visualized as an optical image.

In the apparatus that we developed, the sample was illuminated with CPL, whose purity of polarization was maximized to the greatest possible extent. The present method is based on the idea of discrete illumination of CPL, and the rapid modulation between LCPn and RCPn is enabled to facilitate lock-in detection at the same time. Figure 1b shows a schematic diagram of the discrete modulation of CPL compared with the PEM method of polarization modulation (Fig. 1a). The CPL is obtained by converting LP light with an achromatic quarter waveplate (AQWP05M-600 Thorlabs, Inc.). To maximize the purity of the CPL, high-purity LP light must be provided. To satisfy this requirement, we adopted a polarization beam displacer (BD) (BD40, Thorlabs, Inc.) made of calcite crystal to prepare two spatially separated parallel beams with mutually orthogonal highly pure LPn. The BD optics allows us to obtain a high attenuation ratio of 10^5^:1 between the split pair of orthogonally LP beams. The two orthogonally polarized output beams were separated by 4 mm. A rotatory mechanical chopper designed for the present purpose was utilized to deliver horizontally and vertically polarized beams periodically, and the two beams were sent alternately without temporal overlap. Specifically, a chopper wheel had a 30-slot blade with a 25% duty ratio to prevent the delivery of horizontally and vertically polarized beams at the same moment. By passing through a second BD with exactly the same characteristics as the first one, the two chopped LP beams were recombined as a single beam with the same optical axis. Finally, the quarter waveplate converted the coaxially combined horizontally and vertically polarized beams to LCPn and RCPn, respectively, and the beam was then directed to the sample. The quarter waveplate was adjusted to give equal intensities for linear-polarization components of the circularly polarized light at any polarization direction. As shown in Fig. 2, the modulated light illuminated the sample as an unfocused parallel (collimated) beam to maintain the purity of the circular polarization and avoid the influence of the *z*-components of the electric field. The BD optics can be used in the wavelength region from 350 nm to 2300 nm. To utilize this wide wavelength range, the use of a white (or widely tuneable) light source is preferable. In the present study, we chose a super continuum laser (Super k COMPACT, NKT Photonics A/S) as a broadband collimated light source because it readily provides a stable beam with high brightness and low beam divergence along with efficient energy usage. The white light beam from the laser was monochromatized using a dielectric bandpass filter (10 nm FWHM) and then used for the measurements.

### Microscopic detecting method

In this study, the modulated CPL illuminates the sample as a parallel beam, as described above. To detect the local optical response of the sample, we constructed an imaging system with diffraction optics (i.e., by the use of lenses). Optical images are occasionally acquired directly using an imaging sensor. In the previously reported study of the discrete illumination of CPL, CD images were obtained by determining the difference between LCP and RCP transmission images after integrating many images to reduce the noise level^12^. Although the use of an image sensor such as a charge coupled device (CCD) has an advantage when observing a wide area of a sample at once, standard image sensors are not suitable for use in lock-in detection synchronized with high-speed polarization modulators^13^ because high-speed data acquisition is not easy to perform. In the present study, we adopted a lock-in detection of the signal from a single channel detector for a specific small area to fully utilize the benefits introduced by the high-speed polarization modulation. As shown in Fig. 2, an objective lens (CFI Plan Fluor 40×, Nikon instruments Inc.) with a numerical aperture (NA) of 0.75 provided an approximately 40-times magnified image of the sample, and a pinhole (P100S, Thorlabs, Inc.) was placed on the image plane. Light radiated from an area of interest was selected by the pinhole and detected using a photomultiplier tube (PMT, H10722-20, Hamamatsu Photonics K. K.). We obtained a two-dimensional map of the optical signals (i.e., optical image) by scanning the sample with a closed-loop piezo-driven positioning stage. A diameter of the pinhole at the image plane was 0.1 mm. This pinhole diameter is equivalent to a diameter of approximately 2.5 μm on the sample plane. Reducing the mechanical vibration from the rotatory chopper and suppressing the temperature variation, which changes the properties of optical components, positioning stages and so forth, were essential to achieve long-term stability in the sensitive detection of the signal from the small area. All of the components of the measurement system, except the chopper, were installed on a breadboard with very low mechanical resonance frequency (125BM-8, Minus K Technology Inc.) to avoid any unfavourable effects caused by the vibration of the chopper. The whole measurement system, including the chopper, was fixed on a temperature-regulated optical table (WB-1510T, Nippon Boushin Industry Co., Ltd.) and placed in a box that consisted of doubly heat-insulating walls of aluminum sheets and acrylic boards to ensure thermal stability. The temperature of the optical table was kept constant at 298 K by a water-cooled chiller. With these countermeasures, thermally stable measurements were realized.

### Signal processing and calibration of the CD signal

**Figure 3:**
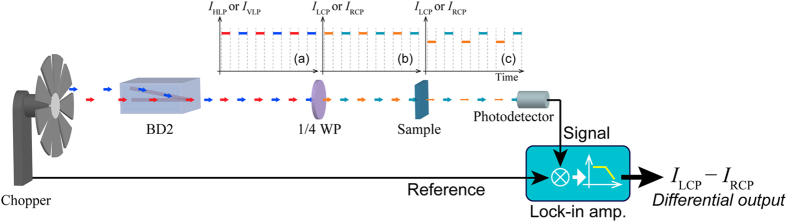
Detection of the differential optical response between LCP and RCP irradiation. In the discrete CPL modulation setup depicted in Fig. 1b, the BD divides an incident LP beam into horizontal LP (HLP) and vertical LP (VLP) components. The HLP and VLP beams are alternately blocked by a chopper blade, which provide light beams that are discretely modulated between two mutually orthogonal linear polarizations (as shown in (**a**), where the red and blue lines represent the HLP and VLP intensities, respectively). The modulated HLP beam and the VLP beam are recombined into a single beam with the same optical axis by BD2 and then converted by a quarter waveplate (1/4 WP) to LCP and RCP light, respectively. The LCP and RCP intensities vary as shown in (**b**), where the orange and green lines represent the LCP and RCP intensities, respectively. The circular-polarization modulated beam transmitted through the sample is detected by a photodetector. If the sample is CD active, then it yields difference in the detected signal intensities between LCPn and RCPn, as shown in (**c**). The differential optical intensity between LCPn and RCPn (*I*_LCP_ − *I*_RCP_) is obtained via lock-in detection.

Signals obtained with a photodetector were sent to a lock-in amplifier. The super continuum laser provided a nanosecond pulse with a repetition rate of 20 kHz at maximum. The laser ignition and the CPL beam modulation with the chopper were synchronized by sharing a signal supplied from a function generator to trigger both the laser and the chopper. Figure 3 shows a sequence of signal processing for the discrete polarization modulation in this work. The chopper blade blocked the two mutually orthogonal LP beams to provide on/off modulation. The frequency of the on/off modulation was set at 200 Hz, which is 1/100 of the (maximum) repetition frequency of the laser. By converting the LP light to CPL via a quarter waveplate, a beam that was modulated between LCPn and RCPn was obtained. Illuminating the sample with this discretely modulated CPL beams enables us to measure the differential extinction (absorbance) (Δ*A* = *A*_L_ − *A*_R_) between LCPn and RCPn, i.e., CD, via the lock-in detection of the optical signals at the chopping frequency.

**Figure 4:**
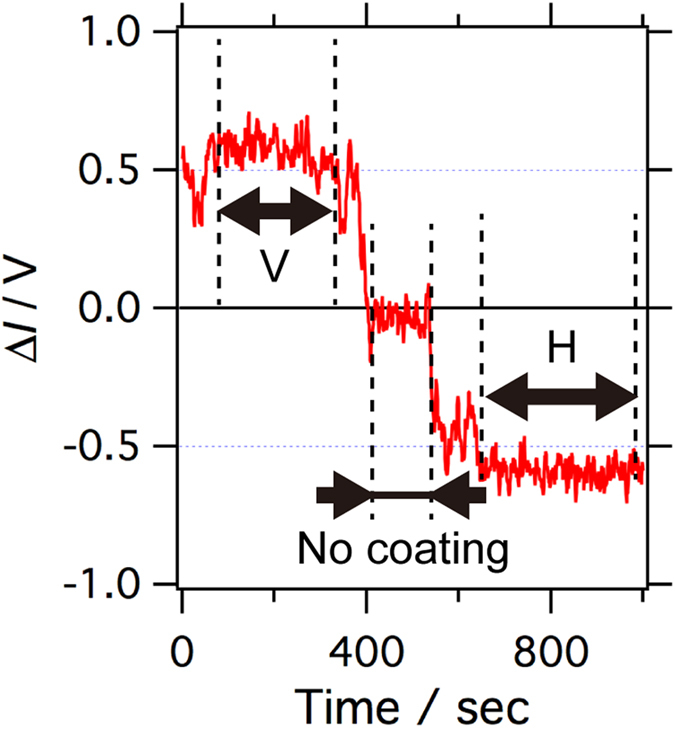
Calibration of the CD signal. The CD signal intensity of the system was calibrated at a wavelength of 700 nm by measuring signal change when a sample with known small absorbance (fused silica substrate whose partial area was coated with Cr; 0.012 OD at 700 nm) was inserted between the two BDs in Fig. 1b. The differential optical intensity (Δ*I*) between the V- and H-beams (see Fig. 1b) when both beams are transmitted through the uncoated area of the calibration sample is referred to as the zero level of the signal. In the time region labelled ‘V’ and ‘H’, the Cr-coated area of the sample attenuated the V-beam and H-beam, respectively. The CD signal intensity was evaluated from the signal change and the known absorbance of the calibration sample. See the main text for more details.

The detected CD signals should be calibrated by measuring the standard chiral molecules in solution, if they are available. However, there is no commonly adopted standard sample that shows a sufficient CD signal in the wavelength range of the present study (500~800 nm); thus, we calibrated the CD signal as described below. We inserted a known sample with a small absorbance into the beam paths between the two BDs (Fig. 1b), where the two beams were linearly polarized, and measured the changes of the output signal from the lock-in amplifier. When one of the two LP beams is attenuated with the sample for calibration placed between the two BDs, the change in the differential signal from the lock-in amplifier is equivalent to the signal change upon the attenuation of either the LCP or RCP beam by the same sample after the quarter waveplate (in the actual experimental setup, the attenuation of one of the circularly polarized beams is not achievable because they are co-axial). As a sample for the calibration with small absorbance, a flat substrate of synthetic fused silica whose surface was partially deposited with a 1-nm-thick uniform Cr layer was used. The absorbance of the sample for calibration in the area coated with the Cr layer was evaluated to be 0.012 in optical density (OD) unit with respect to that of the uncoated area, as determined by spectrophotometric measurement at a wavelength of 700 nm. We measured the relative changes of the signal when the Cr-coated area of the calibration sample was inserted into either the V-beam or H-beam (see Fig. 1(b)), with reference to the control signal, where both V- and H-beams are transmitted through the uncoated area (Fig. 4). The CD signal level was calibrated based on the signal changes obtained above because they correspond to the differential absorbance between LCPn and RCPn. At other wavelengths, the gain of the PMT was adjusted to give the same photocurrent level as that at the calibrated wavelength (700 nm), and the same calibration parameter obtained at 700 nm was used for semi-quantitative measurements. For quantitative measurements, the calibration should be performed at every wavelength.

## Results and Discussion

### Detection limit of the CD signal

The detection limit of the CD measurement obtained using the microscope system was evaluated based on the calibrated CD signal. First, the CD signal was measured at a fixed spot on the surface of the sample for calibration to evaluate the detection limit under no-scanning operation. Because there is no chirality present in the sample, the CD signal must be exactly zero in principle. Next, the standard deviation of temporally fluctuating CD signal (noise level) was estimated. The value obtained gives an approximate value of the detection limit. The standard deviations were 0.0011, 0.000345 and 0.000189 OD under time constants of 30, 300, and 1000 ms for lock-in detection, respectively. These values correspond to 36.3, 11.4, and 6.2 mdeg in the unit of ellipticity, respectively; thus, the detection limit is on the order of 10 mdeg. In the CD measurement system, the super continuum laser is used as a light source. Since white light generation of the laser is based on non-linear optical effect, each laser pulse may have different spectral and polarization features. We evaluated temporal stability of polarization and intensity of the laser output. The laser stability on a typical time scale to acquire a single data point was smaller than ±0.1%. This value is larger than the noise levels arising from the lock-in amplifier and the PMT used in the system, and indicates that the detection limit of the CD measurements performed in this study is determined with the laser stability.

**Figure 5:**
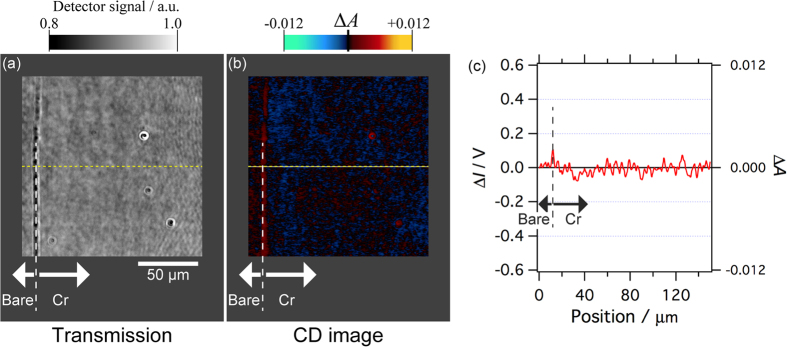
Evaluation of the detection limit of the CD signal. Transmission (**a**) and CD (**b**) images of the fused silica substrate whose partial area was coated with a 1-nm Cr layer. The wavelength of observation was 700 nm; the time constant for lock-in detection was 300 ms. (**c**) Line profile of the CD signal along the dashed lines in (**a**) and (**b**). The standard deviation (noise level) was estimated to be 0.00061 OD.

Next, the detection limit under the imaging measurement was assessed by scanning the same sample to acquire a CD image, as shown in Fig. 5. The CD image acquired with a lock-in time constant of 300 ms showed a nearly zero signal level, except for a region around the boundary between the Cr-coated and bare surface areas. We obtained a standard deviation of 0.00061 OD (20.1 mdeg in ellipticity) from a line profile measured around the middle of the CD image (Fig. 5c). The OD value corresponds to optical absorption of 0.14% (this is still two orders of magnitude poorer than what is expected from the attenuation ratio of 10^5^:1 of the BD optics under an ideal condition). In summary, the system enables the two-dimensional spatial distribution of CD to be visualized as an image if the CD signals are ~20 mdeg or larger in ellipticity. This value was evaluated by using a pinhole (Fig. 2) with a diameter of 0.1 mm. The detection limit may be further improved by increasing the pinhole diameter if obtaining a very high spatial resolution is not necessary.

### CD imaging

**Figure 6:**
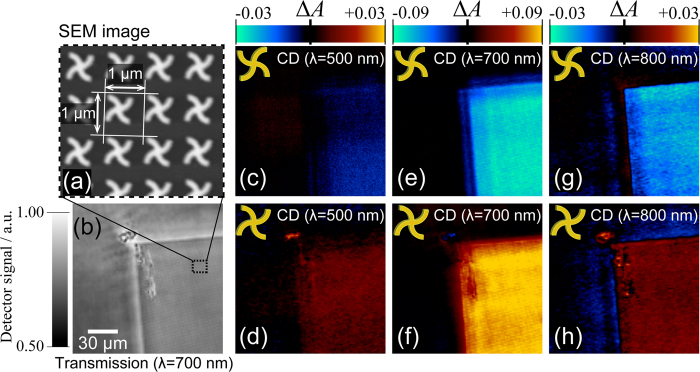
CD images of a sample two-dimensional array of chiral (swirl shape) gold nanostructures. (**a**) SEM image of a swirl-shaped gold nanostructure array. The dimensions of the individual nanostructure are 640 nm (height and width), 160 nm (line width), and 87 nm (thickness). (**b**) Transmission image of the sample at the observation wavelength of 700 nm. (**c**–**h**) CD images observed at wavelengths of 500, 700 and 800 nm. The sample and observation area for (**d**,**f**,**h**) are the same as those for (**b**). The handedness of the chiral nanostructures in panels (**c,e,g**) is reversed to that in (**d,f,h**).

To confirm the ability of the present microscope system to visualize small CD signals with a sufficient spatial resolution, we measured CD images of a two-dimensional chiral metallic nanostructure arrays that were fabricated via electron beam lithography and the lift-off technique. We can fabricate arrays at any area on a substrate with a selected handedness of chirality of the unit structure. As shown in Fig. 6a, we fabricated arrayed swirl-shaped gold nanostructures with a spacing of 1 μm on a glass substrate and measured their CD images. This type of two-dimensional chiral nanostructures has been studied with macroscopic optical measurements by many groups^3^,^14^,^15^. The optical property and mechanism of it are currently discussed based on those results. The total area of the arrayed nanostructures was 150 × 150 μm^2^. Figure 6c–h show the obtained CD images. The handedness of the nanostructures for the images in the upper row (Fig. 6c,e,g) is opposite to that for the lower row (Fig. 6d,f,h). In these images, the CD signals are found to be almost uniform in the areas of the arrayed nanostructures. The CD signals in Fig. 6c and those in Fig. 6d are inverted relative to each other. The inversion of CD signals is also found between Fig. 6e-6f and between Fig. 6g-6h. This result indicates that the observed CD signals correctly reflect the symmetry of the nanostructures.

**Figure 7:**
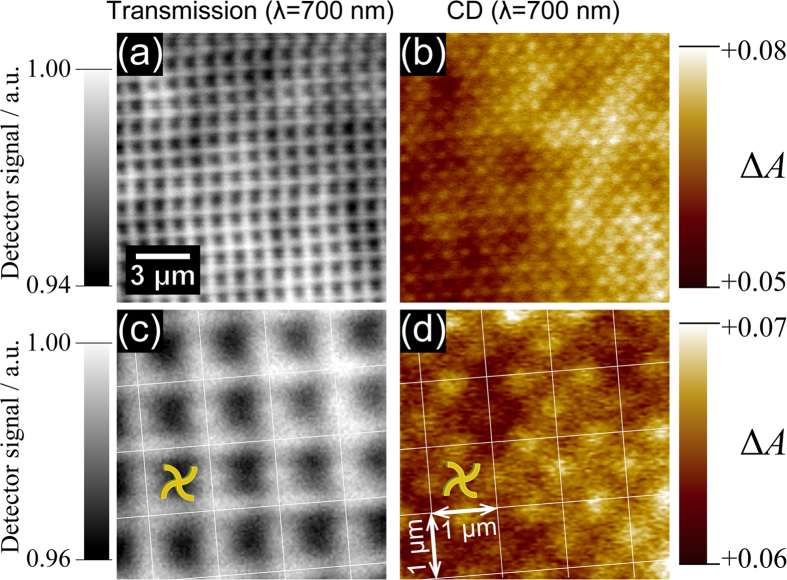
Magnified transmission (**a,c**) and CD (**b,d**) images of the two-dimensional array of chiral (swirl-shaped) gold nanostructures (the same sample as that observed in Fig. 6(b,f)). The wavelength of observation both for the magnified transmission and CD images was 700 nm.

In Fig. 7, a partial area in Fig. 6f is magnified to evaluate the spatial resolution of the CD imaging system. The transmission images (Fig. 7a,c) show the lattice patterns that arise from the arrayed configuration of the nanostructure. As a result of careful comparison between the transmission image and a scanning electron micrograph (SEM) image, we confirmed that the centres of the unit nanostructures were located at the dark (large extinction) regions, as indicated in Fig. 7c. As described in the previous section, a pinhole with a diameter of 0.1 mm was placed on the image plane to detect the local signal, which corresponded to a circular region approximately 2.5 μm in diameter at the objective plane; thus, the signal intensity detected was an average over this region of the sample. This area of the region is several times larger than the unit cell of the nanostructure array with a spacing of 1 μm. In the observed image, each unit cell was clearly resolved with a spatial variation of ~500 nm scale, which is nearly diffraction limited (570 nm, as described below). This observation is apparently contradictory, considering that the pinhole diameter has a larger diameter than the unit cell size at the objective plane. However, it produces an image contrast when an image point of the scanned sample crosses over the pinhole edge due to the detected signal intensity change^16^. The rise of the signal change is determined by the point spread function at the image plane and not by the pinhole diameter. As a consequence, a periodical modulation of the local optical response was observed at a nearly diffraction-limited spatial resolution, which arose from the pitch of the arrayed sample, though the pinhole diameter was larger than the spacing of the array at the image plane. However, note that the image contrast under the use of a 0.1-mm-diameter pinhole is predicted to be reduced to about ten to twenty percent of that obtained when using a sufficiently small pinhole, based on a model simulation. In the model simulation, a square lattice of diffraction-limited point spread functions was assumed to be formed on the image plane, with a lattice constant of 40 μm (the sample lattice constant times the magnification ratio of the imaging system). The optical intensity transmitted through the 0.1-mm-diameter aperture was integrated and then the spatial variation of the integrated intensity on the image plane was evaluated. The reduction of the image contrast was experimentally confirmed for transmission images by comparing the images observed with pinholes with diameters of 0.1 mm and 10 μm (Supplementary information).

In the CD images (Fig. 7b,d), local maximal CD signals are found at four sites at left, right, top, and bottom of the unit nanostructures, rather than at the dark regions in Fig. 7a,c that correspond to the centre of the swirl structures. This fine structure of the CD image reveals that CD is spatially not uniform in the individual unit chiral nanostructure. The spot size of local maximum was 300 to 400 nm (full-width at half-maximum of the peak from the offset level). The separation between the nearest neighbour spots was estimated to be 707 nm. The theoretically predicted spatial resolution of our optical microscope, defined as the minimum separation between two spots that can be resolved on the image (the Rayleigh criterion), is 570 nm for an excitation wavelength of 700 nm. In the CD images, the individual spot separated by a distance of ~700 nm was distinctly resolved, and the spatial resolution of CD imaging was thus apparently improved to the range of 300 to 400 nm. CD is a bipolar signal that can have either a positive or negative value while the minimum signal intensity of the optical extinction or emission image is zero, which is a prerequisite for applying the Rayleigh criterion. When spatial separation between positive and negative CD peak positions is comparable to or smaller than the spatial resolution determined by the Rayleigh criterion, a spatial variation of the CD signal can be steeper than that of the optical extinction or emission. The steeper variation of CD signals may yield the apparent enhancement of the spatial resolution of the CD images, although further discussion is necessary to obtain the details. In the measurements of Fig. 7c,d, the time constant for lock-in detection was 100 ms, and it took approximately 20 minutes to acquire the images with 100 × 100 pixels. For the typical time period required for one CD imaging (~1 hr), temporal variation of the laser was confirmed to be approximately ±0.2% (~0.002 in OD unit). It is not a serious factor that limits the detection sensitivity of the measurements if the observed CD signal is sufficiently large. However, when the signal intensity of the CD image approaches the detection limit, the long-term temporal variation of the laser may slightly shift a base-level of the CD image. In the CD image of Fig. 7d, the Δ*A* value ranges approximately from +0.06 to +0.07 OD. Although the image contrast was only ~0.01 OD, the sub-lattice structures were clearly observed and spatially resolved. The high spatial resolution (300 to 400 nm) and high CD signal sensitivity described above were well balanced, even in the practical CD imaging measurements. In the present set-up, we placed a 0.1-mm-diameter pinhole on the image plane. Thus, an averaged local optical response over the circular region with a diameter of ~2.5 μm at the objective plane was detected, as described above. The contrast of the local CD signal observed here was reduced relative to that of the inherent local CD. The contrast of the CD image will be further enhanced if a pinhole with a smaller diameter is used in combination with a higher intensity light source.

### Difference between CD spectra obtained through microscopic and macroscopic measurements

**Figure 8:**
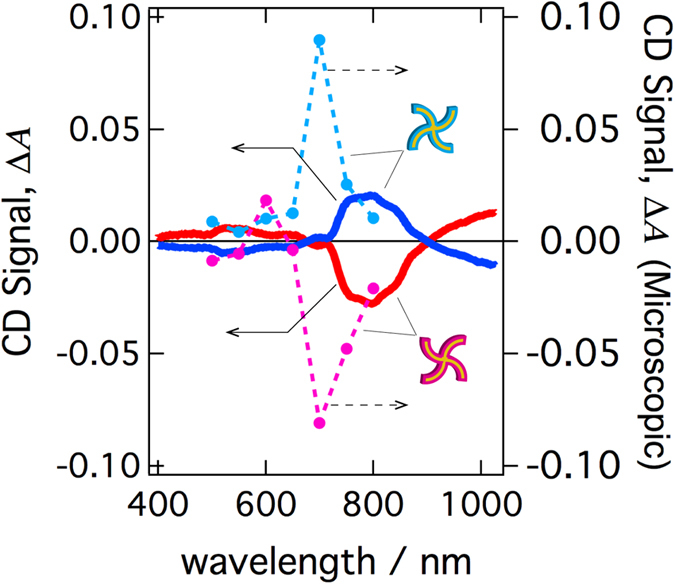
CD spectra of chiral (swirl-shaped) nanostructure arrays. Macroscopic CD spectra measured by a commercial CD spectrometer (solid curves) and CD spectra obtained with the CD microscope (dashed curves). The microscopic CD spectra were obtained by averaging the CD signals in the images over areas of the unit cells of the arrays.

Figure 8 (solid curves) shows the macroscopic CD spectra of the arrayed nanostructures (identical to those investigated above), as measured using a conventional CD spectrometer. The spatial averages of the CD signals obtained by the CD microscope are plotted as a function of wavelength in the same figure (dashed curve). The CD imaging measurement is currently restricted to wavelength regions shorter than 800 nm due to the sensitivity range of the photodetector. At 800 nm, the sign of the CD signal obtained with the microscope was the same as that of the macroscopic CD spectrum, and the amplitude of the CD signal was also in good agreement. In contrast, some differences were found between the signals at shorter wavelengths. The amplitudes evaluated from the microscopic CD signals were much larger than those from the macroscopic CD signals at 700 and 750 nm, and the CD signs were not matched at 500, 550, or 650 nm. In macroscopic spectral measurement by the CD spectrometer, the detected optical responses were obtained from only paraxial rays of transmitted light from the samples because the photodetector was placed far from the sample. In contrast, in the microscopic optical detection system equipped with an objective lens (Fig. 2), radiation was detected over a wide angular range, as determined by the NA number of the lens. The periodical array nanostructures with a 1 μm pitch investigated here may function as an optical diffraction grating. When light is incident normally to a periodic structure with a pitch of *d*, *m*-th diffracted light travels in the direction with an angle *θ*_*m*_, as determined by the diffraction grating equation: *d* sin*θ*_*m*_ = *mλ*. We used an objective lens with an NA of 0.75 for the CD imaging, and in this case, the contribution of diffracted light may be involved, in addition to the normally transmitted light, in the excitation observed at shorter wavelengths than 750 nm. Consequently, differences between the macroscopic and microscopic CD spectra observed at wavelengths shorter than 750 nm may result from the diffraction by the grating structure. In the present CD imaging measurements, diffraction had a significant effect because the samples involved periodic structures. However, diffraction may also affect the signal, even for samples without periodic structures. The contributions from both the absorption component and scattering must be considered for a quantitative treatment of the signal.

We may also need to be careful about birefringence of the objective lens. If an objective lens with very high NA is used, birefringence in the elements constructing the objective lens potentially influence the imaging of scattered light radiated to high NA region. The extraordinary component possibly does not form an image at image plane, and consequently apparent extinction may be overestimated because the extraordinary component is not properly incident onto the photodetector. We measured birefringence of the objective lens used and its dependence on effective NA (pupil size). The result showed that the birefringence was ~1% or less, which indicates that effect on focal length was negligibly small. It was also confirmed that the birefringence of the objective lens was independent of the effective NA.

### Summary

In this work, we constructed a CD microscope that allows the spatial distribution of the CD signals in a sample to be visualized. We employed a method of discretely modulated CPL, where the LP component was excluded and was not incident on the sample. The incident polarization discretely alternates between left- and right-circularly polarized light. The sensitivity and spatial resolution of CD imaging were evaluated by measuring two-dimensional arrays of chiral nanostructures. In the past, CD microscopes were not commonly used because of the difficulties in removing artefacts arising from LP anisotropy that were superimposed on a genuine CD signal due to imperfect polarization modulation. Instead of a PEM device installed in most of the CD spectrometers, the use of a Pockels cell as an alternative modulation device to provide modulated CPL was reported^17^. However, note that Pockels cells generally have larger strain and optical distortion than do those used for PEMs^7^,^8^. The approach proposed in this study demonstrated that highly sensitive and reliable microscopic CD measurement that is less influenced by LP anisotropy is achievable, and nearly diffraction-limited spatial resolution was realized in the practical microscopic observation. By using this approach, chirality analysis based on optical activity signal, to clarify chiral domain structures and/or distribution of chirality involved, is now available, even for anisotropic samples (such as crystalline materials) that have not been considered when using the conventional polarization modulation technique. The proposed CD microscope may be a convenient tool for analysing the handedness of microcrystalline samples or for imaging the chirality distributions in polycrystalline, amorphous, and liquid crystal samples.

The evaluated spatial resolution of CD imaging was apparently even higher than that of a common microscope. Unlike optical intensity signals that give zero or larger values, CD is a differential signal that gives both positive and negative values, which potentially leads to a steeper spatial variation than that of the optical intensity. Consequently, the CD images may provide apparently sharper spots, and the spatial resolution may be enhanced. We previously demonstrated near-field CD imaging with a spatial resolution of ~100 nm by detecting the CD response of the sample under the illumination of polarization modulated light^9^,^18^,^19^,^20^ (in those studies, a PEM was employed to modulate the light polarization). The near-field approach of CD imaging provides us with a valuable method for investigating the nanoscopic structures of CD signals and exploring chiral functionality and its origins in nanostructures and molecular assemblies. By replacing the microscopic detection part of the present experimental apparatus with a near-field optical microscope, a high-sensitivity near-field CD microscope can be realized; one such microscope is currently under development. Although near-field microscopy is advantageous for measurements of static samples, it is less suitable for the live imaging of biological systems and the real-time observation of systems that undergo chemical reactions or transport because it is based on a scanning probe microscope that requires a long acquisition time. Although the CD microscope constructed in the present work requires a moderately long acquisition time to obtain a CD image, it would be possible to investigate the temporal changes of CD images for slowly varying samples. If a high-speed video camera were installed in a detection system of our CD microscope, the apparatus would be applicable to live-imaging.

Compared with other advanced microscopic methods, such as Raman scattering microscopy^21^,^22^, CD-based imaging methods have not been utilized as frequently, partly because of the difficulties in eliminating the artefacts associated with the LP components for anisotropic samples. The sensitivity and spatial resolution achieved for CD imaging in this study will make this imaging method a unique approach for the live observation and tracking of chiral molecules and of the chirality transitions caused by the formation of an aggregate/assembly. This new methodology for the observation and quantitative analysis of the chirality of materials may be applied in a wide range of research areas in the medical and biological fields. The method will also be useful for the chirality analysis of microcrystals. If an imaging technique regarding CD as a probe signal is well established, then a new live bio-imaging field will emerge. Such an imaging technique may contribute to, for example, the identification of chirality of chromatin structures in biological cells^23^,^24^. Recently, the biological activities of D-amino acids have been reported. As an example, the enzymatic conversion of pre-existing L-amino acid to D-amino acid was studied^25^. The use of a CD microscope may provide essential information on the handedness inversion and the transport process that occur in biological cells. The approach proposed in the present study must have great advantages for local CD detection in these investigations.

## Additional Information

**How to cite this article**: Narushima, T. and Okamoto, H. Circular Dichroism Microscopy Free from Commingling Linear Dichroism via Discretely Modulated Circular Polarization. *Sci. Rep.* **6**, 35731; doi: 10.1038/srep35731 (2016).

## Supplementary Material

Supplementary Information
