# Supplementary material for: Circular Dichroism Microscopy Free from Commingling Linear Dichroism via Discretely Modulated Circular Polarization
Source: Sci Rep. 2016 Oct 20;6:35731. doi: 10.1038/srep35731 (PMC5071757; doi:10.1038/srep35731)
Supplement: Supplementary Information [file srep35731-s1.pdf]

Supplementary information

Circular Dichroism Microscopy Free from Commingling Linear Dichroism via Discretely Modulated Circular Polarization

Tetsuya Narushima[AB] and Hiromi Okamoto[A*]

[A]Institute for Molecular Science and The Graduate University for Advanced Studies (Sokendai), 38 Nishigonaka, Myodaiji, Okazaki, Aichi 444-8585, JAPAN

[B] PRESTO, Japan Science and Technology Agency, 4-1-8 Honcho, Kawaguchi, Saitama, 332-0012, JAPAN

* E-mail: aho@ims.ac.jp

Correlation between microscopic optical image contrast and the diameters of the pinhole placed at the image plane

Microscopic optical images in this study were formed by illuminating samples with collimated light and detecting optical responses from the samples through an objective lens (CFI Plan Fluor 40X, Nikon instruments Inc.). The optical response at a position on the sample was extracted through a pinhole placed at the image plane. We obtained an optical image (i.e., a two-dimensional map of the local optical response) by scanning the sample. The period of the unit cell in the arrayed sample observed was 1 μm, which corresponds to 40 μm (= 40 (magnification of the objective lens) × 1 μm (period)) at the image plane. The pinhole diameter (100 μm) looks not enough (too large) to resolve each unit cell of the arrayed nanostructure as a sample image, although we clearly observed the periodic structure in the experimental image (Fig. 7). In the following we describe a model analysis on spatial variation of the optical intensity in the microscopic image, observed with a large pinhole.

As shown in Fig. S1a, optical field distribution of the arrayed structure was modeled by placing a point source at each position of the unit nanostructure, whose image at the image plane was assumed to be described with an Airy function. Fig. S1b shows distribution of optical field intensity at the image plane formed with the arrays of Airy functions. The optical intensity distribution of the array (Fig. S1b) was numerically integrated over the area of the pinhole aperture, to obtain a quantity that corresponds to the observed intensity at a position of the pinhole. This quantity was evaluated at each position of measurement along the scanning line, which gives a simulated line profile of optical intensity observed through the aperture (Fig. S2). In Fig. S2, we have found that periodic variation of the optical signal arising from the arrayed nanostructure is observable even when a 100-μm diameter pinhole is used, although the image contrast is not high. The contrast ratio of 90 % obtained with a 10-μm diameter pinhole was reduced to ~3 % for that with a 100-μm diameter pinhole.

Figure S3 shows experimentally observed transmission images obtained with the measurement system presently developed, for the arrayed nanostructure sample (the same sample used for CD imaging in the main text). Periodic variation in optical intensity with an image-contrast ratio of ~3 % was observed for a 100-μm diameter pinhole (Figs. S3(b,d)), which is comparable to the results obtained with the model simulation (Fig. S2(c)). In contrast, high image-contrast (~35 %) was also confirmed for the measurement

with a 10-μm diameter pinhole.

As described above, it has been confirmed, both with the model simulation and the experimental measurement, that periodic variation in optical intensity, which reflects the arrayed structure of the sample, is observable even when a large aperture pinhole is placed at the image plane, although the image contrast is reduced.

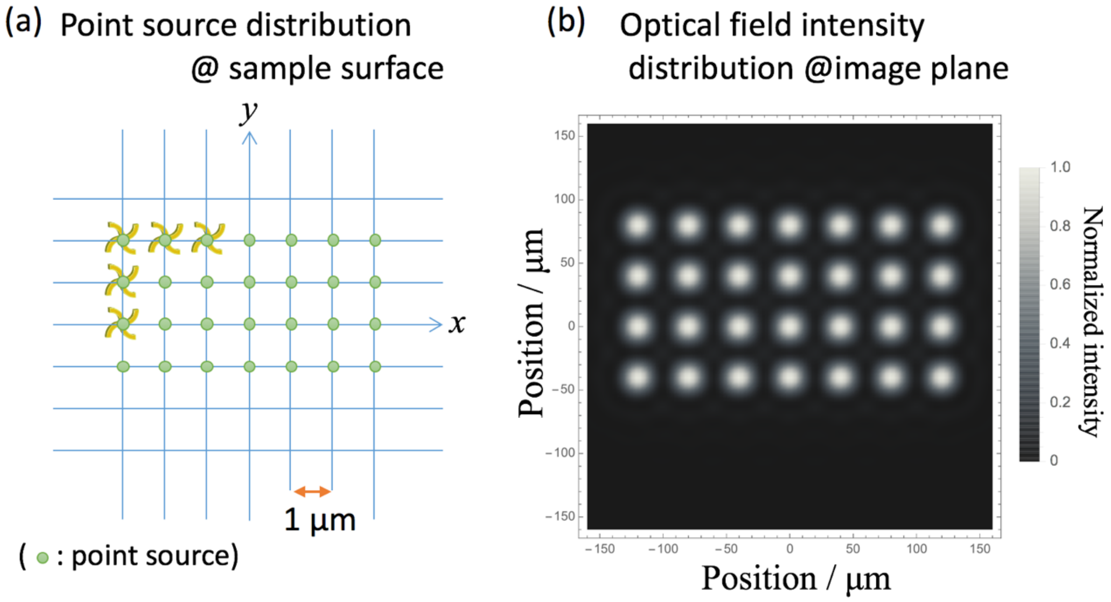

Fig. S1 A model for optical field distribution of the arrayed structure. (a) Point light sources (represented with dots) are placed at positions of unit nanostructures on the sample surface. (b) Optical field intensity distribution at the image plane formed with an ensemble of Airy functions. The wavelength of the simulation was 700 nm.

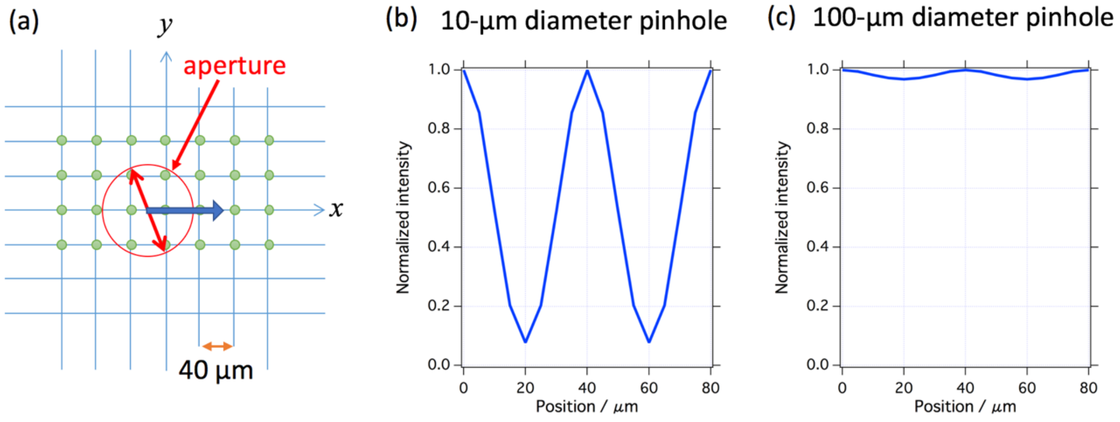

Fig. S2 Simulated line profiles of optical field intensity passing through the pinhole. (a) Scan trajectory (blue arrow), along lattice points of the point sources. (b, c) Line profiles of the optical field intensities through 10- and 100-µm diameter pinholes along the scan trajectory indicated in panel (a).

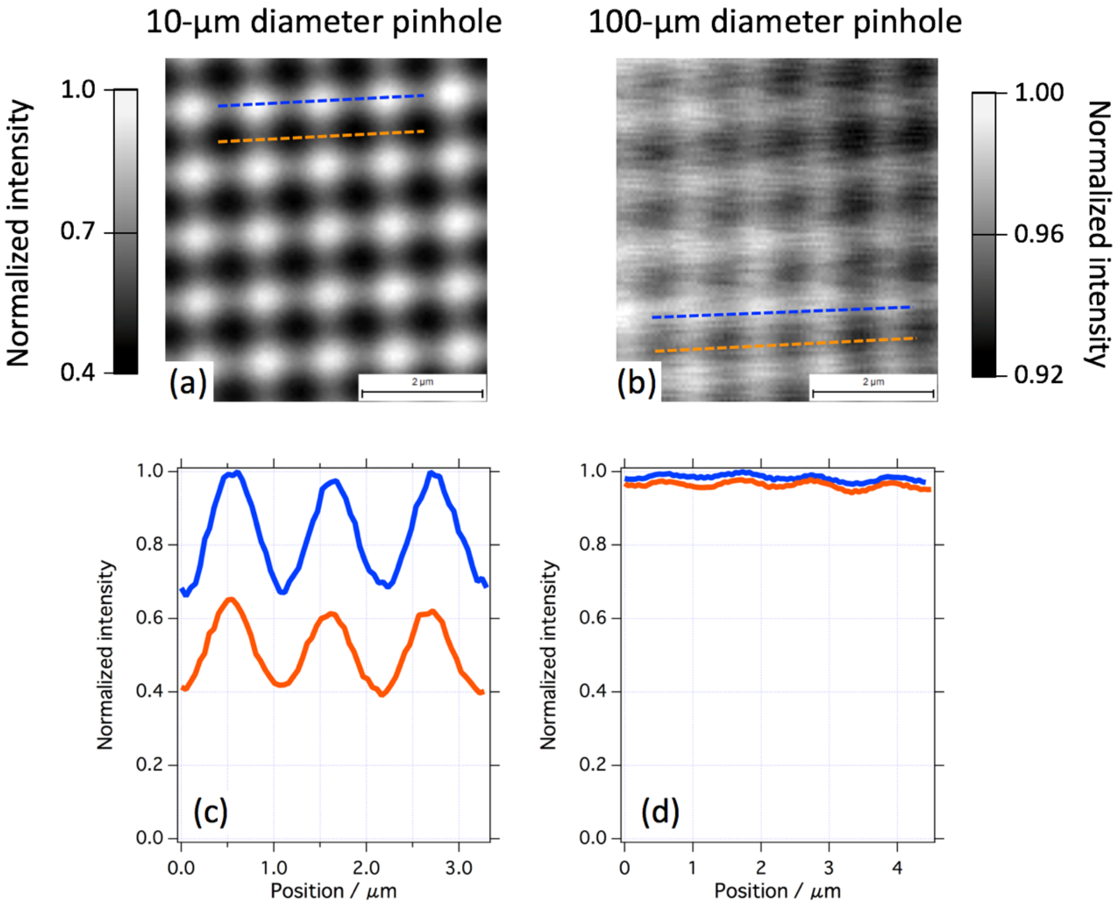

Fig. S3 (a, b) Transmission images experimentally obtained with pinholes of 10- and 100-μm diameters. (c, d) Normalized line profiles of optical intensity obtained along dashed lines in panels (a) and (b). The wavelength of observation was 700 nm.
